# Can body mass index, waist circumference, waist-hip ratio and waist-height ratio predict the presence of multiple metabolic risk factors in Chinese subjects?

**DOI:** 10.1186/1471-2458-11-35

**Published:** 2011-01-13

**Authors:** Yong Liu, Guanghui Tong, Weiwei Tong, Liping Lu, Xiaosong Qin

**Affiliations:** 1 Clinical laboratory, Shengjing Hospital of China Medical University, Shenyang 110004, PR China

## Abstract

**Background:**

Obesity is associated with metabolic risk factors. Body mass index (BMI), waist circumference, waist-hip ratio (WHR) and waist-height ratio (WHtR) are used to predict the risk of obesity related diseases. However, it has not been examined whether these four indicators can detect the clustering of metabolic risk factors in Chinese subjects.

**Methods:**

There are 772 Chinese subjects in the present study. Metabolic risk factors including high blood pressure, dyslipidemia, and glucose intolerance were identified according to the criteria from WHO. All statistical analyses were performed separately according to sex by using the SPSS 12.0.

**Results:**

BMI, waist circumference and WHtR values were all significantly associated with blood pressure, glucose, triglyceride and also with the number of metabolic risk factors in both male and female subjects (all of P < 0.05). According to receiver operating characteristic (ROC) analysis, the area under curve values of BMI, waist circumference and WHtR did not differ in male (0.682 vs. 0.661 vs. 0.651) and female (0.702 vs. 0.671 vs. 0.674) subjects, indicating that the three values could be useful in detecting the occurrence of multiple metabolic risk factors. The appropriate cut-off values of BMI, waist circumference and WHtR to predict the presence of multiple metabolic risk factors were 22.85 and 23.30 kg/m2 in males and females, respectively. Those of waist circumference and WHtR were 91.3cm and 87.1cm, 0.51 and 0.53 in males and females, respectively.

**Conclusion:**

The BMI, waist circumference and WHtR values can similarly predict the presence of multiple metabolic risk factors in Chinese subjects.

## Background

Obesity is associated with metabolic risk factors such as high blood pressure, blood fat abnormality, and glucose intolerance, which may influence the morbidity and mortality of cardiovascular diseases [1-4]. Body mass index (BMI) is the most widely used indicator of weight status and has been applied into both public health and clinical practice. However BMI does not consider the distribution of body fat, resulting in variability in different individuals and populations [5]. Waist circumference, waist-hip ratio (WHR) and waist-height ratio (WHtR) are used to predict the risk of obesity related diseases as they account for regional abdominal adiposity [6-8].There are studies reporting that both BMI and waist circumference values can equally identify cardiovascular risk factors [9-11]. The American Diabetes Association has stated that it's not clear whether WC can predict cardiovascular risk factor better that BMI [12]. Suggesting that there are some controversial issues around the adiposity marker that better predicts cardiovascular risk factors.

It is also known that the relation between BMI and percentage body fat is influenced by age, sex, and ethnicity [13-15]. In some Asian populations, a higher percentage of body fat was found for a given BMI than that in Europeans [16,17]. Nevertheless, the relationship between obesity indicators such as BMI and waist circumference and cardiovascular risk factors has not been fully established in Chinese population. Moreover, the association between these four indicators and the clustering of cardiovascular risk factors has not been studied.

In this study, BMI, waist circumference, WHR and WHtR values were compared to predict the occurrence and clustering of cardiovascular risk factors in Chinese subjects.

## Methods

The present study included 772 Chinese subjects in Liaoning Province, China, and had an annual health check-up during the period between year 2008 and 2009. The nonrepresentative convenience samples were selected from communities in Shenyang City, Chaoyang City, Huanren County, Qingyuan County, Yingkou City and Wafangdian City in Liaoning Province. The human investigations were approved by the Institutional Review Board of Shengjing Hospital of China Medical University, and the written informed consents were obtained from all subjects.

Duplicate measures of height, weight, hip circumference (at the level of maximal gluteal protrusion) and waist circumference (at the midpoint between the anterior superior iliac crest and the lowest rib) were obtained by trained researchers using standard techniques [18]. BMI was calculated as weight (kg) divided by squared height (m^2^), WHR and WHtR were determined from waist circumference (cm) divided by hip circumference (cm) and height (cm), respectively. Blood pressure was recorded in duplicate after 5 min of rest by using random-zero sphygmomanometers. Fasting blood samples were obtained for measurement of glucose, lipids, and lipoproteins by using standard techniques. The subjects were instructed to fast - nothing to eat or drink except water - 12 hours before taking the blood samples. The blood samples were taken from the vein

Metabolic risk factors were diagnosed based on the definition released by World Health Organization for the Diagnostic Criteria of Metabolic Syndrome: 1) high blood pressure; systolic blood pressure ≥140 mmHg and/or diastolic blood pressure ≥90 mmHg, 2) dyslipidemia; triglyceride ≥1.695 mmol/l and/or HDL-C <40 mg/dL, and 3) glucose intolerance; fasting plasma glucose ≥5.6 mmol/l. Two or more risk factors were defined as "multiple" risk factors.

All statistical analyses were performed separately according to sex by using the Statistical Package for Social Science (SPSS version 12.0). P values of less than 0.05 were considered to indicate statistical significance. The clinical and biochemical data of the study subjects were expressed as means ± SD. The differences between two groups were examined by t-test or ANOVA for the continuous variables and by χ2-test for the categorical variables. Receiver operating curve (ROC) analyses were used to determine the appropriate values for four indicators according to male and female. The appropriate point was defined as the closest point on the ROC curves to the point at 1-specificity of 0 and sensitivity of 100%.

## Results

Characteristics of the study population are shown in Table 1. There were 360 males and 412 females in this study. Male subjects had significantly greater waist circumference, height and weight than female subjects. WHtR value was significantly smaller in males than in females. BMI and WHR were similar in two groups. Metabolic profiles also differed; male subjects had higher systolic and diastolic blood pressure (significantly), fasting glucose (significantly), and triglyceride concentration but lower total cholesterol values than female subjects.

**Table 1:** Characteristics of the study population

Characteristics	Total	Males	Females	P value
Number of subjects	772	360	412	
Age (years)	49.47 ± 16.53	49.68 ± 16.93	49.30 ± 16.19	0.749
Height (cm)	162.37 ± 10.17	167.91 ± 9.03	157.54 ± 8.51	<0.001
Weight (kg)	64.17 ± 13.24	68.17 ± 13.10	60.67 ± 12.36	<0.001
Waist circumference (cm)	88.90 ± 11.61	89.90 ± 10.78	88.02 ± 12.23	0.024
Body mass index (kg/m2)	24.27 ± 4.70	24.04 ± 3.73	24.48 ± 5.39	0.187
Waist to hip ratio	0.96 ± 0.16	0.97 ± 0.13	0.96 ± 0.17	0.437
Waist to height ratio	0.55 ± 0.07	0.54 ± 0.06	0.56 ± 0.08	<0.001
Systolic blood pressure (mmHg)	128.31 ± 20.29	128.81 ± 18.48	127.87 ± 21.76	0.520
Diastolic blood pressure (mmHg)	83.27 ± 11.81	84.21 ± 11.09	82.45 ± 12.35	0.039
Fasting glucose (mmol/l)	5.69 ± 1.57	5.82 ± 1.90	5.58 ± 1.20	0.040
Triglyceride (mmol/l)	1.53 ± 1.21	1.60 ± 1.26	1.47 ± 1.16	0.142
Total cholesterol (mmol/l)	4.88 ± 1.04	4.81 ± 0.90	4.94 ± 1.16	0.090

All values are means ± SDs.

Table 2 shows the relationship between BMI, waist circumference, WHR, WHtR and blood pressure, fasting glucose, triglyceride in males and females separately. Elevated BMI, waist circumference and WHtR were apparent in high blood pressure, fasting glucose and triglyceride groups, respectively (all of P values were less than 0.05). However the relationship was not seen for WHR.

**Table 2:** BMI, waist circumference, WHR and WHtR values according to the different metabolic risk factors in male and female subjects

	Male				Female			
	**BMI**	**waist circumference**	**WHR**	**WHtR**	**BMI**	**waist circumference**	**WHR**	**WHtR**

Systolic blood pressure								
<140 mmHg	23.59 ± 3.72	88.39 ± 11.03	0.96 ± 0.14	0.53 ± 0.06	23.83 ± 5.71	85.73 ± 12.07	0.95 ± 0.18	0.54 ± 0.08
≥140 mmHg	25.34 ± 3.46	94.29 ± 8.67	0.98 ± 0.11	0.56 ± 0.05	26.11 ± 4.10	93.80 ± 10.67	0.98 ± 0.14	0.60 ± 0.07
P value	<0.001	<0.001	0.369	<0.001	<0.001	<0.001	0.070	<0.001
Diastolic blood pressure								
<90 mmHg	23.37 ± 3.64	87.95 ± 10.78	0.96 ± 0.14	0.53 ± 0.06	23.88 ± 5.49	86.31 ± 11.96	0.96 ± 0.18	0.55 ± 0.08
≥90 mmHg	25.44 ± 3.53	93.98 ± 9.60	0.98 ± 0.11	0.56 ± 0.06	26.28 ± 4.69	93.15 ± 11.65	0.96 ± 0.13	0.59 ± 0.08
P value	<0.001	<0.001	0.242	<0.001	<0.001	<0.001	0.641	<0.001
High blood pressure								
No	23.24 ± 3.71	87.36 ± 11.02	0.96 ± 0.14	0.52 ± 0.06	23.72 ± 5.81	85.41 ± 12.14	0.95 ± 0.19	0.54 ± 0.08
Yes	25.23 ± 3.45	93.71 ± 9.21	0.98 ± 0.12	0.56 ± 0.05	25.80 ± 4.27	92.64 ± 11.00	0.97 ± 0.14	0.59 ± 0.07
P value	<0.001	<0.001	0.255	<0.001	<0.001	<0.001	0.429	<0.001
Fasting glucose								
<5.6 mmol/l	23.60 ± 3.58	89.11 ± 10.71	0.98 ± 0.15	0.53 ± 0.06	23.88 ± 5.30	86.47 ± 12.31	0.96 ± 0.19	0.55 ± 0.08
≥5.6mmol/	24.81 ± 3.93	91.39 ± 10.82	0.95 ± 0.11	0.55 ± 0.06	25.63 ± 5.55	90.90 ± 11.61	0.95 ± 0.14	0.58 ± 0.08
P value	0.003	0.055	0.009	0.018	0.002	0.001	0.493	<0.001
Triglyceride								
<1.695 mmol/l	23.34 ± 3.64	88.40 ± 11.19	0.97 ± 0.154	0.53 ± 0.06	23.76 ± 5.73	85.33 ± 11.88	0.95 ± 0.19	0.54 ± 0.08
≥1.695 mmol/l	25.78 ± 3.50	93.84 ± 8.70	0.96 ± 0.10	0.56 ± 0.06	26.51 ± 4.09	95.55 ± 9.91	0.98 ± 0.14	0.61 ± 0.07
P value	<0.001	<0.001	0.775	<0.001	<0.001	<0.001	0.090	<0.001

Abbreviation: BMI, Body mass index (kg/m2). WHR, Waist to hip ratio. WHtR, Waist to height ratio.

All values are means ± SDs.

**Figure 1:**
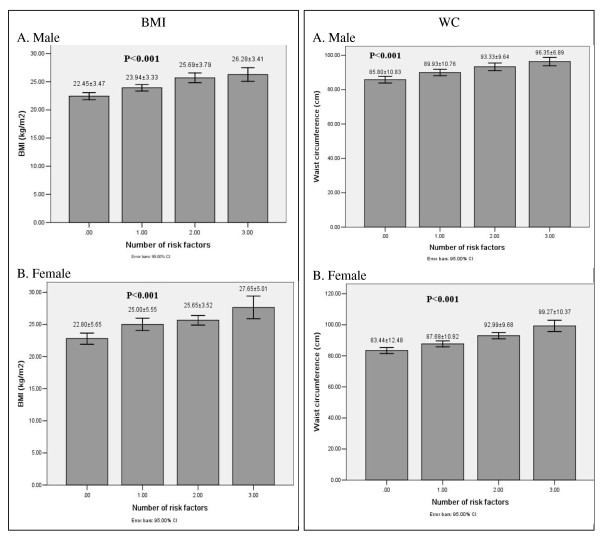
**BMI and WC values according to the number of metabolic risk factors in male (A) and female (B) subjects**.

**Figure 2:**
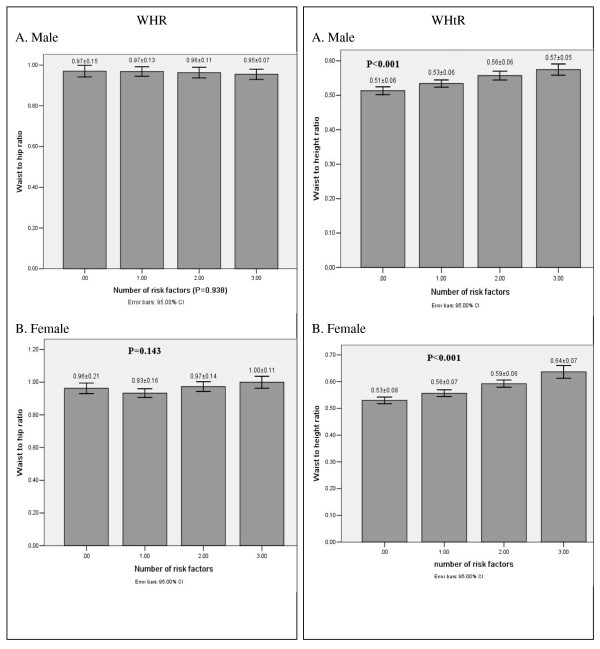
**WHR and WHtR values according to the number of metabolic risk factors in male (A) and female (B) subjects**.

Figure 1 and 2 shows the relationship between BMI, waist circumference, WHR, WHtR values and the numbers of metabolic risk factors. The BMI, waist circumference and WHtR values were all significantly greater according to the increase in the numbers of metabolic risk factors in both males and females. But the relationships were not significant between WHR and the numbers of metabolic risk factors.

**Figure 3:**
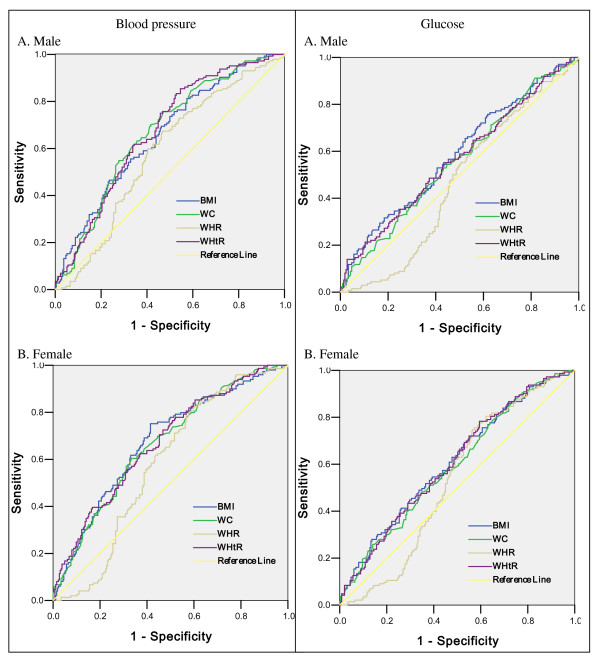
**The ROC (receiver operating characteristic) curves for BMI, WC, WHR and WHtR values to detect high blood pressure and glucose in male (A) and female (B) subjects**. AUC: area under curve

**Figure 4:**
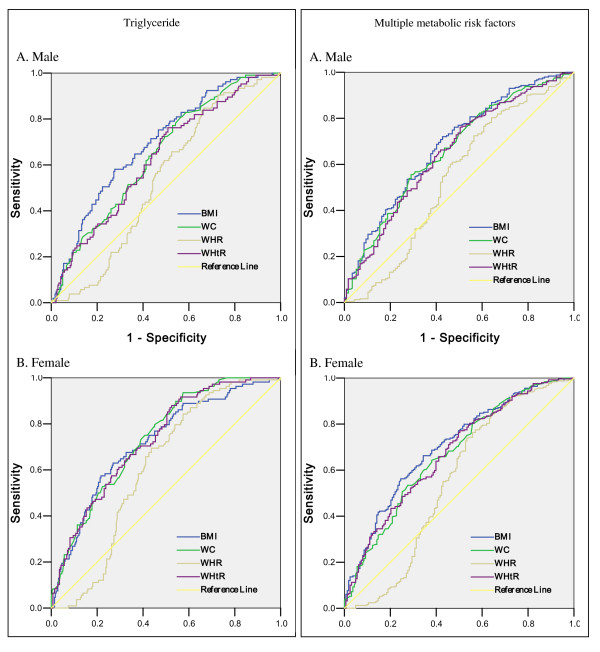
**The ROC (receiver operating characteristic) curves for BMI, WC, WHR and WHtR values to detect high triglyceride and multiple metabolic risk factors in male (A) and female (B) subjects**. AUC: area under curve

Figure 3 and 4 showed the ROC curves to determine the appropriate BMI, waist circumference, WHR and WHtR values for detecting the presence of high blood pressure, fasting glucose, triglyceride and multiple risk factors in males and females, respectively. In male subjects with the cutoff value of 23.00kg/m^2 ^(for BMI), 89.05cm (for waist circumference),0.92 (for WHR), 0.51 (for WHtR), the sensitivity and specificity were 76% and 49%, 70% and 42%, 67% and 54%, 85% and 46%, respectively, which were found to be the cut-off values to detect high blood pressure. The cutoff values for fasting glucose were 22.41kg/m^2 ^(for BMI), 90.75cm (for waist circumference), 0.92 (for WHR), 0.54 (for WHtR), and the corresponding sensitivity and specificity were 76% and 40%, 46% and 56%, 61% and 46%, 55% and 57%, respectively in males. The cut-off values to detect high triglyceride were 24.93kg/m^2 ^for BMI (sensitivity and specificity were 58% and 73%), 86.55cm for waist circumference (sensitivity and specificity were 81% and 44%), 0.88 for WHR (sensitivity and specificity were 85% and 37%), 0.52 for WHtR (sensitivity and specificity were 76% and 50%). The cut-off values to detect multiple risk factors in males were 22.85kg/m^2 ^(for BMI), 91.30cm (for waist circumference), 0.87 (for WHR), 0.51 (for WHtR), and the corresponding sensitivity and specificity were 72% and 58%, 45% and 71%, 80% and 36%, 76% and 50%, respectively. In female subjects with the cutoff value of 23.30kg/m^2 ^(for BMI), 90.90cm (for waist circumference),0.85 (for WHR), 0.54 (for WHtR), the sensitivity and specificity were 75% and 59%, 60% and 67%, 83% and 40%, 78% and 48%, respectively, which were found to be the cut-off values to detect high blood pressure. The cutoff values for fasting glucose were 22.87kg/m^2 ^(for BMI), 81.50cm (for waist circumference), 0.87 (for WHR), 0.53 (for WHtR), and the corresponding sensitivity and specificity were 69% and 47%, 79% and 45%, 75% and 44%, 78% and 41%, respectively in females. The cut-off values to detect high triglyceride were 25.00kg/m^2 ^for BMI (sensitivity and specificity were 63% and 74%), 82.75cm for waist circumference (sensitivity and specificity were 93% and 43%), 0.86 for WHR (sensitivity and specificity were 84% and 43%), 0.54 for WHtR (sensitivity and specificity were 88% and 48%). The cut-off values to detect multiple risk factors in females were 23.30kg/m^2 ^(for BMI), 87.1cm (for waist circumference), 0.86 (for WHR), 0.53 (for WHtR), and the corresponding sensitivity and specificity were 66% and 66%, 64% and 62%, 74% and 47%, 77% and 50%, respectively.

Table 3 shows the area under curve (AUC) values of BMI, waist circumference, WHR and WHtR by using ROC analysis to detect the presence of high blood pressure, fasting glucose, triglyceride and multiple risk factors in both sexes. AUC values did not differ between BMI, waist circumference and WHtR in either males or females.

**Table 3:** Area under Curves of BMI, waist circumference, WHR and WHtR for Multiple Risk Factors

	BMI	waist circumference	WHR	WHtR
Male				
Blood pressure	0.657 (0.601-0.713)*	0.669 (0.613-0.725)*	0.582 (0.523-0.641)*	0.673 (0.617 ± 0.728)*
Glucose	0.586 (0.525-0.647)*	0.557 (0.495-0.618)	0.468 (0.408-0.529)	0.567 (0.505 ± 0.629) *
Triglyceride	0.693 (0.635-0.752)*	0.646 (0.586-0.706)*	0.536 (0.475-0.597)	0.631 (0.569 ± 0.693)*
Multiple metabolic risk factors	0.682 (0.623-0.741)*	0.661 (0.601-0.722)*	0.539 (0.470-0.608)	0.651 (0.590 ± 0.712)*
Female				
Blood pressure	0.674 (0.621-0.728)*	0.666 (0.613-0.719)*	0.579 (0.524-0.633)*	0.671 (0.618 ± 0.724)*
Glucose	0.610 (0.552-0.667)*	0.594 (0.537-0.652)*	0.532 (0.475-0.589)	0.607 (0.550 ± 0.664)*
Triglyceride	0.721 (0.665-0.777)*	0.738 (0.687-0.788)*	0.611 (0.556-0.667)*	0.731 (0.679 ± 0.783)*
Multiple metabolic risk factors	0.702 (0.651-0.754)*	0.671 (0.617-0.724)*	0.552 (0.490-0.614)	0.674 (0.621 ± 0.727)*

All values were AUC (95%CI).

AUC, area under curves; CI, confidence interval.

*P < 0.05

## Discussion

The present study suggested that waist circumference and WHtR as well as BMI values were equally useful indicators to identify the presence of multiple cardiovascular risk factors in Chinese subjects. The cut off values of BMI to predict multiple cardiovascular metabolic risk factors were 22.85 kg/m2 and 23.30 kg/m2 in males and females, respectively. Those of waist circumference and WHtR were 91.3cm and 87.1cm, 0.51 and 0.53 in males and females, respectively.

The World Health Organization provided guidelines for classifying body weight status based on BMI and demonstrated a close relation between BMI and cardiovascular risk factors [17]. Recently, waist circumference and waist circumference -related values has been widely used as a representative indicator of abdominal adiposity, because they are correlated with abdominal fat mass and are more associated with cardiovascular risk factors than BMI [19-21]. However, the efficiency of these two indicators to detect the presence of cardiovascular risk factors has been controversial [6-12]. The present study suggested that both BMI and waist circumference, also its related WHtR might identically predict the presence of multiple metabolic risk factors in Chinese population.

The World Health Organization Western Pacific Region suggested the cutoff value of obesity as BMI≥25kg/m^2 ^in the Asia-Pacific region [22]. A study indicated the optimal cutoff points for BMI with regard to the presence of at least 2 metabolic risk factors were lowest in East Asians (24kg/m^2^) and suggested uniform anthropometric cutoff values for all Asian ethnic groups are not appropriate to assess obesity-related metabolic complications [23]. Nguyen [24] found that optimal BMI cutoffs were 23-24, 21-22.5, and 20.5-21 for Chinese, Indonesian, and Vietnamese adults, respectively. Thus, the appropriate BMI cut-off values to detect the presence of multiple metabolic risk factors in Chinese population may be lower than 25 kg/m^2^. The result of this study was similar with that.

Miyawaki et al [25] demonstrated that the appropriate cutoff waist circumference values were 86cm for males and 77cm for females to detect multiple risk components by using Japanese criteria based on their visceral-fat area cut-off levels of 100cm2 in males and 65cm2 in females. A study in Korean population suggested that the optimal waist circumference values were 84-86 cm for men and 78-80 cm for women to detect multiple cardiovascular risk factors [26]. Ko et al [27] determined that in Chinese population waist circumference of 84.6cm in men and 75.7cm in women were the optimal cutoff values to predict high mesenteric fat thickness with ROC analysis. The appropriate cut-off values of waist circumference in our study were higher than those of these previous studies.

Whether specific values measuring central fat distribution could more accurately indicate health risk than BMI remains a controversial issue [28-30]. WHtR has received considerable interest and the result suggested keeping one's waist to less than half his height [31-33]. A Chinese study reported that waist to stature ratio (WSR) (or saying waist to height ratio) is the best simple anthropometric indicator in predicting a wide range of cardiovascular risk factors and related health conditions. They analyzed 11 cardiovascular risk factors in partial correlation analysis, including ties WSR had the highest r in 6 in men, and 5 in women; followed by waist circumference with 4 in men and 6 in women. In ROC analyses of 21 risk factors and health conditions, the area under curve (AUC) of WSR was the largest for most (13 of 21) factors in men and 10 in women. The optimal WSR cutoff value was 0.48 for both men and women. [33]. In our analyses, WHtR cutoff values were 0.51 and 0.53 in males and females, respectively. However, in the present study WHR was not significantly increased among subjects with multiple risk factors as well as WHR did not present an AUC significance (in the ROC analysis) to predict the presence of cardiovascular risk factors. The reason may be the sample of this study can not representative of the adult population of China or the sample size is not big enough. It is possible that similar analyses undertaken in a representative sample would yield different estimates.

Takahashi et al [34] demonstrated that combining of both waist circumference and BMI was superior to using only one of these parameters. Wang et al suggested that both BMI and waist circumference, rather than waist circumference alone, should be included in metabolic risk assessment in this high-risk multiethnic Asian population. Uniform anthropometric cutoff values for all Asian ethnic groups are not appropriate to assess obesity-related metabolic complications [23]. In the present study, BMI, waist circumference, WHR and WHtR were analyzed together to predict multiple metabolic risk factors in males and females. However, in this study the accuracy of anthropometric variables as indicators of Multiple Metabolic Risk was not high. Swets [35] suggested that the 0.5 < AUC < 0.7 indicates that the diagnostic is less accurate. Further studies are needed to evaluate the association between these four values and future occurrence of cardiovascular events to define their appropriate cut-off values in Chinese population.

## Conclusions

The present study suggested that BMI, waist circumference and WHtR values were all associated with metabolic risk factors, and they may equally predict multiple metabolic risk factors. Although our conclusions might be one of the important instructions for public health promotion to maintain appropriate BMI, waist circumference and WHtR values by lifestyle modification including diet and exercise, this would have been impractical given the nature and size of the study population.

## Competing interests

The authors declare that they have no competing interests.

## Authors' contributions

The authors' responsibilities were as follows—YL, GT, and WT: developed the idea and wrote the first and subsequent versions of the manuscript; LL and XQ: completed all statistical analyses; and YL, and GT: were principal and coinvestigators on various projects and contributed data and to the writing of the manuscript. All authors read and approved the final manuscript.

## Pre-publication history

The pre-publication history for this paper can be accessed here:

http://www.biomedcentral.com/1471-2458/11/35/prepub
